# Ketamine As A New Therapeutic Option For The Management Of Mental Disorders

**DOI:** 10.1192/j.eurpsy.2022.2277

**Published:** 2022-09-01

**Authors:** M. Turki, O. Abidi, S. Ellouze, R. Ben Jmeaa, J. Nasri, N. Halouani, J. Aloulou

**Affiliations:** Hedi Chaker University Hospital, Psychiatry “b” Department, Sfax, Tunisia

**Keywords:** Ketamine, Mental Disorders, resistant

## Abstract

**Introduction:**

Ketamine is routinely used for anesthetic induction because of its dissociative properties. Recently, it has attracted attention as a rapid-acting anti-depressant, but other studies have also reported its efficacy in the management of diverse psychiatric pathologies previously resistant to treatment.

**Objectives:**

We aimed to review the efficacity of ketamine in the management of mental disorders.

**Methods:**

We conducted a litterature review through pubmed database, using the following keywords: “mental illness”; “ketamine”;”depression”;”anxiety disorders”;”eating disorders”;”substance use disorders”.

**Results:**

Ketamine has primarily been used in psychiatry for people with treatment-resistant depression. Its efficacy in reducing suicidal ideation has been previously reported. Furthermore, Ketamine may be a potential therapeutic option for patients with treatment-resistant anxiety disorders, especially obsessive compulsive disorder (OCD) and post-traumatic stress disorder. It has recently been reported a rapid onset anxiolytic activity in treatment-resistant social anxiety disorder and generalized anxiety disorder. Besides, Ketamine use in subjects suffering from eating disorders was associated with a complete remission of severe anorexia nervosa with a return to normal weight and a decrease in body preoccupations. The use of ketamine alone or in combination with other therapies was effective in reducing alcohol and substance use, prolonging abstinence, reducing craving and enhancing motivation. ketamine in combination with motivational enhancement therapy may be an effective pharmacotherapy for initiating and sustaining abstinence from alcoholics

**Conclusions:**

ketamine shows great promise as a treatment for several mental disorders. However, its possible side effects and short duration of efficacy limit its use. Further studies exploring longer-term outcomes and administration protocols are needed.

**Disclosure:**

No significant relationships.

